# Relationship Between Hardiness and the Mental Health of Funded Chinese College Students: The Mediating Role of Social Support and the Moderating Role of an Only-Child Status

**DOI:** 10.3389/fpsyg.2022.842278

**Published:** 2022-03-24

**Authors:** Jingxuan Liu, Xiaoshuang Cheng, Jun Li

**Affiliations:** ^1^Media and Communication College, Yunnan Normal University, Kunming, China; ^2^Department of Education Management, Chinese International College, Dhurakij Pundit University, Bangkok, Thailand

**Keywords:** funded college students, impoverished college students, hardiness, mental health, the status of only-child/non-only-child, social support

## Abstract

According to the hardiness model and the perspective of different treatment by parents, this study developed and validated a moderated mediation model to explore the direct effect of hardiness on the mental health of Chinese funded college students (FCSs), the mediating role of social support, and the moderating role of only-child (OC) /non-only-child (NOC) status. A hardiness scale, mental health scale, and perceived social support scale were used to examine information on 673 Chinese FCSs. Hardiness had a significantly positive effect on the mental health of FCSs. Mediation analysis indicated that social support mediated the relationship between hardiness and the mental health of FCSs. The moderated mediation model analysis indicated that the OC/NOC status moderated the second half of the mediation model. The results suggest that the hardiness model is applicable to FCSs from China and elucidate the internal influence mechanism between hardiness and mental health. On the basis of the findings of this study, suggestions are presented in this paper for college education management.

## Introduction

Compared with regular Chinese college students, funded college students (FCSs) often face financial difficulties for reasons such as familial poverty and they are funded by various national financial aid sources from Chinese government, society, and other different channels (Ministry of Education of the PRC and Ministry of Finance of the PRC, 2007; The State Council of the PRC, 2007). Since China implemented the Targeted Poverty Alleviation Strategy (TPAS), the Chinese government has increased financial support for college students in poverty. However, several studies have demonstrated that their mental health is generally worse than that of economically stable Chinese college students because of upbringing and personal factors (Hu, 2010; Wang et al., 2015; Cheng et al., 2021). In addition, since the COVID-19 outbreak, educational and living environments have changed worldwide, and Chinese college students have been challenged by problems such as depression, anxiety, poor interpersonal communication skills, and difficulties in adaptation (Bai et al., 2021; Jin et al., 2021; Liu et al., 2021; Pan et al., 2021). Empirical studies have indicated that the prevalence of mental health problems among college students from economically disadvantaged families has increased because of the pandemic (Liu et al., 2021). Therefore, exploring methods for improving the mental health of FCSs is crucial.

Studies have indicated that numerous complex factors affect college students’ mental health. Mental health is often affected by a combination of personality traits, family-related factors, education, social support, and other environmental factors (Liang, 2004; Dias and Cadime, 2017; Lai, 2021; Li et al., 2021; Wei et al., 2021). Positive personality traits such as optimism, resilience, and calmness can mitigate the negative effects on mental health under stressful circumstances (Maddi and Khoshaba, 1994; Achat et al., 2000; Hong and Xu, 2009). Studies have suggested that hardiness can positively affect college students’ mental health (Ramzi and Besharat, 2010) and reduce negative emotions such as anxiety caused by academic stress (Hystad et al., 2009; Oktavia et al., 2019; Abdollahi et al., 2020). Hence, hardiness may have a significant positive effect on the mental health of FCSs.

According to the hardiness model (Maddi, 2002), hardiness can help individuals process the physical and mental distress caused by stress by acquiring social support. The model explains how hardiness affects physical and mental health through social support (Maddi and Khoshaba, 1994; Maddi, 2002; Kinder, 2005). Research has indicated that hardiness positively affects individuals perception of social support (Ganellen and Blaney, 1984; Du and Chai, 2014) and that hardy individuals receive more social support than do those who are not hardy and can therefore adapt to academic and psychological challenges (Du and Chai, 2014). The hypothetical model of social support explains that social support not only directly improves mental health but also mitigates the negative effects of stress on physical and mental health (Barrera, 1986; Lee et al., 2004; Wang, 2004; Yang, 2016; Zhang et al., 2018). Social funding and support are more crucial for college students with financial difficulties because they can relieve the pressure caused by financial difficulties and help students establish healthy interpersonal relationships to reduce psychological distress (Hefner and Eisenberg, 2009; Zhao, 2017; Cheng et al., 2021). Therefore, social support may play a mediating role in the effect of hardiness on the mental health of Chinese FCSs.

The one-child policy (OCP) is a unique national policy in China. Individuals growing up as only-children (OCs) or non-only-children (NOCs) may receive different treatment and social support. The OC families got positive attitudes, privileges, and financial subsidy (Chu et al., 2015; Wang, 2016). In contrast, NOC families were less socially acceptable (Chu et al., 2015). Although the OCP has retired from the historical stage currently, the two groups of OCs and NOCs still co-exist in China (Li et al., 2018). Previous research has reported a negative relation between quantity and quality of children per family because of the conflict between parents’ living level and that of their children (Becker and Lewis, 1973). For the impoverished students’ families, social support is an effective way for to relieve financial stress (Zheng, 2021). Besides, the perspective of different treatments by parents suggest OCs may differ from NOCs in individual’s adaptability, behavioral patterns, and physical and mental health (Falbo and Polit, 1986; Kolm and Ythier, 2006; Minuchin, 2018; Wang and Yuan, 2019). Thus, this study focuses on the moderating effect of OC on the relationship between social support and mental health.

Although studies have explored the relationships among hardiness, social support, and college students’ mental health, few have investigated specific groups of Chinese FCSs, who have been reported with more apparent mental health problems in previous studies (Cheng et al., 2021; Liu et al., 2021) and require a high level of attention. Therefore, this study used social support as a mediating variable to explore the internal influence mechanism of hardiness on the mental health of Chinese FCSs. And considering the difference of perceived social support between OCs and NOCs, this study focuses on whether OC/NOC status moderates the relationship between social support and mental health.

## Literature Review

### Hardiness and Mental Health

Kobasa (1979) introduced hardiness into the field of psychology as a positive personality trait for processing stress. On the basis of existential psychology, hardiness includes commitment, control, and challenge, and it is defined as attitudes, beliefs, and behaviors that mitigate the negative effects of life events and help individuals process and avoid physical and mental distress (Kobasa, 1979; Maddi, 2002). Hardy individuals usually exhibit strong foresight and control in response to environmental changes, display effective coping behaviors, and believe that they can influence their surroundings through their efforts and thereby turn crises into opportunities for growth rather than passively accepting the consequences of these crises (Kobasa, 1979; Kobasa et al., 1981; Maddi, 2002). Studies have found that hardiness can mitigates the negative health effects of academic stress (Hystad et al., 2009; Abdollahi et al., 2020). Studies have also observed that hardiness reduces anxiety, improves social skills, and explains discrepancies in college students’ mental health (Sadeghi and Einaky, 2020). These findings indicate that hardiness is essential for college students to maintain their physical and mental health throughout their academic careers and lives.

Mental health is a state of wellbeing in which individuals have positive inner experiences and high social adaptability and can develop their potential in their work, studies, and life (Liang, 2004; Zhang et al., 2013). The hardiness model explains how hardiness can lead individuals to protect themselves against psychological and physical distress through several methods (Maddi, 2002; Kinder, 2005). Research has shown that hardiness is significantly and positively correlated with numerous positive personality traits, significantly and negatively correlated with stress and negative coping behaviors, and a predictor of mental health (Eschleman et al., 2010). Hardy individuals have a strong ability to process academic stress (Oktavia et al., 2019), which mitigates its negative effects on their health (Hystad et al., 2009). Hardiness is essential for college students to maintain their mental health (Maddi and Khoshaba, 1994; Yong, 2017). Ramzi and Besharat (2010) discovered that hardiness has a significant and positive effect on the training and mental health of college athletes and enhances athletic performance. Financially disadvantaged Chinese college students may face more pressure, obstacles, and challenges to their mental health throughout their studies and lives than does the general student population. On the basis of the aforementioned discussion, the following hypothesis (Hypothesis 1) is proposed as: hardiness is a significant positive predictor of the mental health of FCSs.

### Mediating Role of Social Support

Social support refers to various types of assistance provided by an individual’s social system and the care, attention, and respect that they receive from members of society (Barrera, 1986; Malecki and Demary, 2002; Cheng et al., 2021). Research has indicated that students’ perception of social support can enhance their subjective wellbeing (Yan et al., 2011; Liu et al., 2016), sense of meaning in life, and mental health (Wei et al., 2021).

The hardiness model indicates that hardy individuals can maintain their physical and mental health through social support, positive coping strategies, and healthy behaviors. This model also suggests that the relationship between hardiness and mental health is mediated by social support (Maddi and Khoshaba, 1994; Maddi, 2002; Kinder, 2005). Research has indicated that personality factors affect an individual’s access to and perception of social support (Wang, 2004), that the hardiness of college students is significantly and positively related to social support (Ganellen and Blaney, 1984), and that hardiness and social support lead to a positive perception of subjective wellbeing (Duan, 2010). Hardiness significantly and positively predicts college students’ perception of social support and affects their adaptability to school through social support, which suggests that students with high levels of hardiness can use social resources to protect themselves from stress, physically and mentally adapt to the academic environment, and adjust their role in this environment (Du and Chai, 2014).

Studies have explored three hypothetical models on the relationship between social support and mental health: the main effect model, buffering model, and dynamic model (Barrera, 1986; Wang, 2004). The main effect model indicates that social support has a direct and positive effect on physical and mental health. According to this model, the higher the level of social support, the better is an individual’s physical and mental health. Liu (2020) suggested that improving social support systems during the COVID-19 pandemic can directly improve adolescents’ mental health. Perceived social support has a lasting effect on the development of college students’ mental health (Zhang et al., 2018). Social support has a complete mediating effect on the relationship between parenting style and the mental health of financially challenged college students and can directly predict their mental health (Yang, 2016). The buffering model demonstrates the role of social support in maintaining and improving physical and mental health by mitigating the negative effects of stress. Studies have revealed that social support improves subjective wellbeing by counteracting depression and increasing self-control (Yan et al., 2011), that social support to impoverished Chinese college students indirectly affects their mental health through mediating variables (Cheng et al., 2021), and that social support can mitigate the stress caused by the process of adjusting to an international academic environment and thus affect mental health (Lee et al., 2004). The dynamic model suggests that social support, psychological stress, and mental health have a complex interacted relationship that changes over time and depending on the environment, as evidenced by Monroe and Steiner (1986). The aforementioned three models (and aforementioned cited studies) reveal the effects of social support on mental health.

For Chinese FCSs, in addition to personal characteristics, support from social systems is a key environmental variable that determines their success in higher education and in maintaining their physical and mental health. Social support can relieve economic pressure and enable students to feel care from their communities. Therefore, hardiness was hypothesized to affect mental health through social support. Consequently, the following hypothesis (Hypothesis 2) is proposed: social support has a mediating role in the relationship between hardiness and the mental health of FCSs.

### Moderating Role of OC/NOC Status

OC and NOC families exist in China (Li et al., 2018). The parenting styles and upbringing in OC and NOC families affect individuals’ adaptive capacity, behavior, and physical and mental health differently (Falbo and Polit, 1986; Kolm and Ythier, 2006; Minuchin, 2018; Zheng et al., 2019). Research has identified that differences exist in personality, character, and resources between individuals from OC and NOC families (Falbo and Polit, 1986; Cameron et al., 2013; Wang and Yuan, 2019). OCs may lack communication skills, an ability to cooperate (Minuchin, 2018), and an awareness of competition and display a strong tendency toward risk aversion (Cameron et al., 2013; Wang and Yuan, 2019). However, OCs may have strong leadership skills (Smith, 1984), a motivation to achieve, and intellectual skills (Falbo and Polit, 1986).

One study discovered differences in parenting styles between OC and NOC families. Chinese impoverished college students from OC families feel considerably more emotional warmth and understanding than do those from NOC families (Zhang, 2014). Previous studies found family was one of the key environment factors on individual’s personalities and mental health (Lai, 2021; Wei et al., 2021). But for Chinese FCSs, social support plays a critical role in relieving poverty and protecting mental health (Cheng et al., 2021). Thus, this study proposes the following hypothesis (Hypothesis 3): the status of OC/NOC moderates the second half of the mediating relationship between hardiness and the mental health of FCSs through social support.

**Figure 1:**
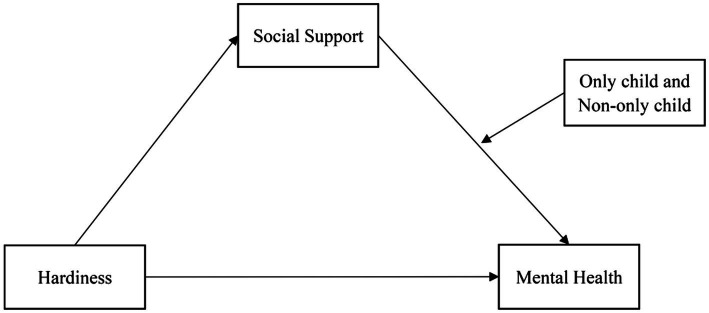
Hypothetical model.

Figure 1 presents a synthesis of the theoretical analysis and literature review into a hypothetical model.

## Materials and Methods

### Participants

Yunnan province, which is located at the southwest border of China, is a key target area of the TPAS and a particularly impoverished region. This study used purposive sampling to recruit 673 participants from Yunnan’s officially identified FCSs group. The FCS identified work was carried out ahead by official committees of each department in the college based on documents by the government, in which standards or requirements about family background of FCSs are set (Ministry of Education of the PRC and Ministry of Finance of the PRC, 2007; The State Council of the PRC, 2007). The participants were informed of the purpose of this study through an online survey and consented to participate. They were informed that they were free to withdraw from the survey at any point. A total of 684 questionnaires were distributed, of which 11 were deemed invalid and excluded for extremely short response times and missing answers; thus, 673 valid questionnaires remained, which resulted in a recovery rate of 98%. A total of 135 participants (20.1%) were men, and 538 participants (79.9%) were women. A total of 122 participants (18.1%) were OC, and 551 participants (81.9%) were NOC. The sample ranged in age from 18 to 23.

### Measures and Procedure

First, the descriptive statistics of the sample were obtained, and a correlation analysis, scale reliability test, and common method deviation (CMV) test were performed using SPSS. Confirmatory factor analysis (CFA) was performed using AMOS. Second, Model 4 in PROCESS was used to test the mediation effect, Model 14 was used to test the moderated mediation effect, and bootstrap confidence intervals were used to determine whether the mediating effect from Model 4 and moderating effect from Model 14 were significant (Hayes, 2017).

#### Hardiness Scale

This study used the hardiness scale for Chinese college students developed under the unique Chinese sociocultural context by Lu and Liang (2008), which was tested with good reliability and validity in previous empirical studies (Lu and Liang, 2008; Chen and Tu, 2019). The scale comprises four dimensions, namely, control, challenge, input, and resilience, and 27 questions that are scored using a 5-point Likert scale. An example of the items in this scale is “I often regard the difficulties encountered in life as challenges rather than as threats.” The Cronbach’s α of the aforementioned scale was 0.96. CFA indicated that the standardized factor loadings were between 0.67 and 0.84, which are greater than 0.5 (Fornell and Larcker, 1981; Bagozzi and Yi, 2012). The aforementioned result indicates that the adopted hardiness scale had high reliability and validity. The model fit indices were as follows: *χ*^2^/df = 3.52, RMR = 0.03, RMSEA = 0.06, CFI = 0.94, GFI = 0.88, NFI = 0.91, TLI = 0.93, and PNFI = 0.83. These results indicate ideal fit (Bollen, 1989; Schumacker and Lomax, 2004).

#### Perceived Social Support Scale

This study adopted the Multidimensional Scale of Perceived Social Support developed by Zimet et al. (1988), which comprises 12 items related to three dimensions, namely, support from family, support from friends, and support from a significant other. These items are rated on a 7-point Likert-type scale. An example of the aforementioned items is “There is a special person who is around when I am in need.” The Cronbach’s α of the aforementioned scale was 0.94, and CFA indicated that the standardized factor loadings were between 0.82 and 0.88, which are greater than 0.5. This result indicates that the aforementioned scale had high reliability and validity. The model fit indices were as follows: *χ*^2^/df = 4.77, RMR = 0.03, RMSEA = 0.07, CFI = 0.97, GFI = 0.94, NFI = 0.96, TLI = 0.96, and PNFI = 0.75. These results indicate ideal fit (Bollen, 1989; Schumacker and Lomax, 2004).

#### Mental Health Scale

This study used the mental health scale developed by Zhang et al. (2013), which comprises six items in one dimension that are rated on the 5-point Likert scale. An example of these items is “I enjoy my life.” The last item in this scale was reverse-scored, and CFA indicated that its factor loading was lower than 0.5; thus, this item was removed (Kline, 2010). The Cronbach’s α of the aforementioned scale was 0.91, and the standardized factor loadings were between 0.78 and 0.84, which are greater than 0.5. This result indicates that the aforementioned scale had high reliability and validity. Because this scale is unidimensional, a multifactor oblique intersection model was used to test its fit indicators with respect to the other two scales, and the results indicated satisfactory fit (Table 1).

**Table 1:** Model fit indices.

Standard	X^2^/df	RMR	RMSEA	CFI	GFI	NFI	TLI	PNFI	HOELTER.05<0.08
	<5	<0.08	<0.08	>0.9	>0.85	>0.9	>0.9	>0.5	>200
Results	2.74	0.03	0.05	0.93	0.85	0.90	0.93	0.83	266

#### CMV Test

Harman’s one-factor test was used to test for CMV. Unrotated factor analysis revealed that the Kaiser–Meyer–Olkin value was 0.97 (>0.8). Significant results were obtained in Bartlett’s test of sphericity (*p* < 0.001). The explanatory power of the first factor was 38.06%, which is lower than the 50% threshold (Podsakoff et al., 2003); thus, CMV was not a significant problem in this study.

## Results

### Descriptive Statistics of and Correlation Between the Investigated Variables

Table 2 presents the descriptive statistics of hardiness, social support, and mental health. Correlation analysis revealed that hardiness and the mental health were significantly and positively correlated, with a correlation coefficient of 0.61 (*p* < 0.001). Hardiness and social support were significantly and positively correlated, with a correlation coefficient of 0.57 (*p* < 0.001). Social support and the mental health were significantly and positively correlated, with a correlation coefficient of 0.64 (*p* < 0.001). The correlation coefficients between any two of the three variables were lower than 0.8, which indicates that low-to-moderate correlation existed between the variables, and no collinearity was observed (Benesty et al., 2009).

**Table 2:** Descriptive statistics of and correlation between the investigated variables.

Variable	*M*	*SD*	Hardiness	Social support	Mental health
Hardiness	3.85	0.61	1		
Social support	3.89	0.76	0.57^***^	1	
Mental health	4.02	0.77	0.61^***^	0.64^***^	1

*N = 673*.

*** *p < 0.001.*

### Mediating Role of Social Support

Model 4 of PROCESS was used to test the mediating effect of social support. Table 3 presents the results of this test. In Model 1, hardiness significantly and positively predicted the mental health (*B* = 0.77, *p* < 0.001). In Model 2, hardiness significantly and positively predicted social support (*B* = 0.71, *p* < 0.001). When social support was used as a mediating variable in Model 3, hardiness significantly and positively predicted the mental health of the *B* = 0.46, *p* < 0.001; however, the predictive power of hardiness in Model 3 was weaker than that in Model 1. Social support was a significant and positive predictor of the mental health of *B* = 0.44, *p* < 0.001, which suggests that social support partially mediates the effect of hardiness on mental health. Bias-corrected nonparametric percentile bootstrapping was conducted to test the mediating effect of social support. An indirect effect value of 0.31 and a 95% confidence interval (CI) of 0.24–0.38, which does not include 0 was obtained. The mediating effect accounted for 40.41% of the total effect, which indicates the significance of the mediating effect of social support.

**Table 3:** Results obtained in the testing of the mediation of social support.

	Model 1	Model 2	Model 3
Variable	Mental health	Social support	Mental health
	B (*t*)	B (*t*)	B (*t*)
Hardiness	0.77(19.86^***^)	0.71(17.94^***^)	0.46(10.87^***^)
Social support			0.44(12.97^***^)
*R^2^*	0.37	0.32	0.50
*F*	394.38^***^	321.75^***^	330.36^***^

*B represents unstandardized coefficients*.

*** *p < 0.001.*

### Moderated Mediation Model

**Figure 2:**
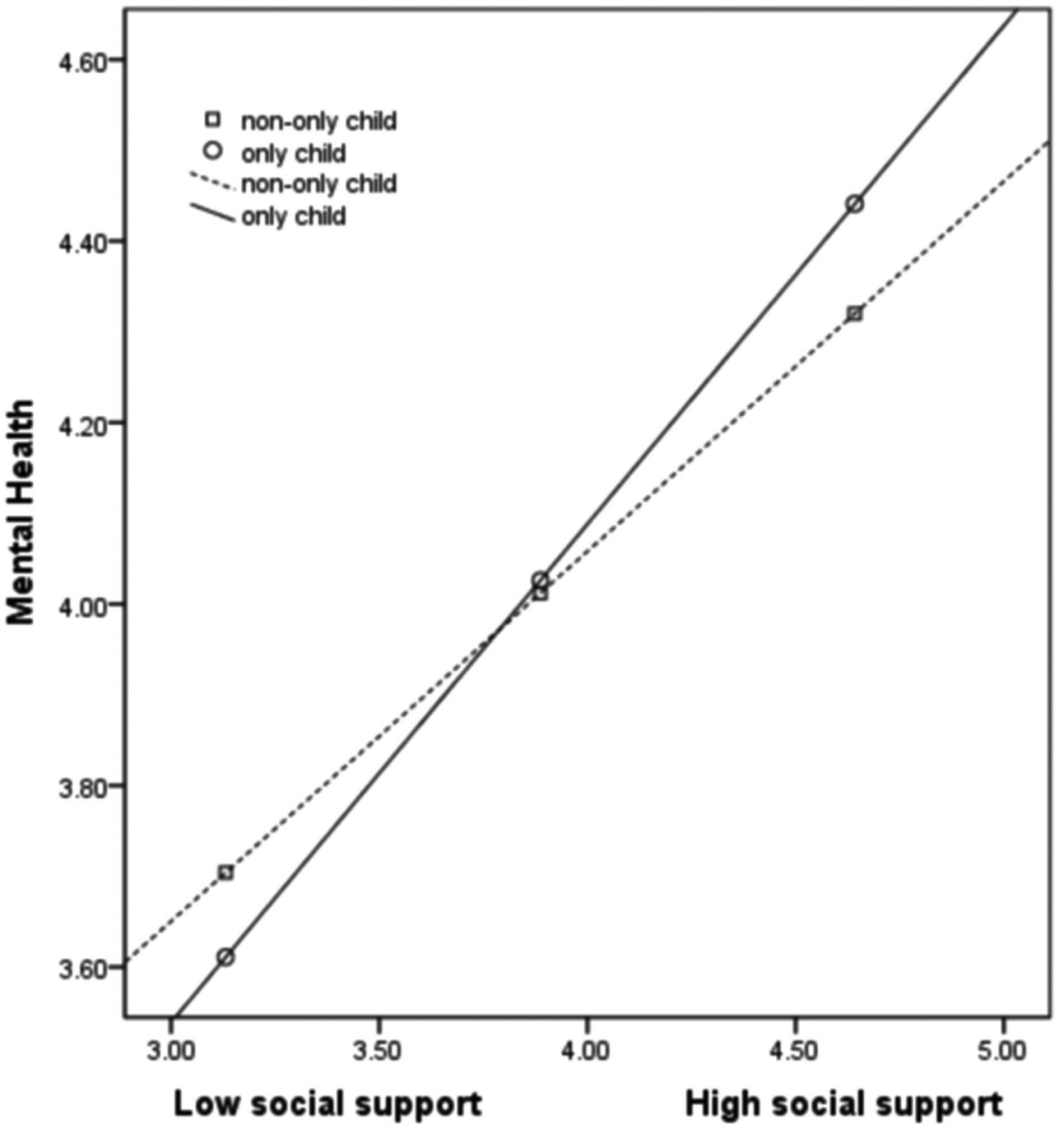
Moderating effect of only-child/non-only-child status on the relationship between social support and mental health.

Model 14 of PROCESS was used to determine whether the mediation effect was moderated by the OC/NOC status (Table 4). Hardiness was a significant predictor of social support and mental health, with social support being a significant predictor of mental health in Model 2 (*B* = 0.41, *p* < 0.001). The interaction of social support and the OC/NOC status was a significant predictor of mental health (*B* = 0.14, *p* < 0.05), which indicates that the OC/NOC status moderated the second half of the mediation in the effect of hardiness on the mental health through social support. This finding was verified through bias-corrected nonparametric percentile bootstrapping. The indirect effect of hardiness on mental health through social support was moderated by the OC/NOC status, with the index of moderated mediation being 0.10 (lower-limit CI = 0.01, upper-limit CI = 0.18) and the CI not containing 0, which indicates that the moderated mediation model is valid. Thus, the moderated mediation was stronger for FCSs who are OCs (*B* = 0.39, lower-limit CI = 0.29, upper-limit CI = 0.47) than for those who are NOCs (*B* = 0.29, lower-limit CI = 0.23, upper-limit CI = 0.35). Simple slope analysis (Figure 2) indicated that social support was a stronger predictor of mental health for FCSs who are OC (simple slope = 0.55, *t* = 9.07, *p* < 0.001) than for FCSs who are NOCs (simple slope = 0.41, *t* = 10.92, *p* < 0.001).

**Table 4:** Results obtained in the testing of the moderated mediation model.

	Model 1	Model 2
Variable	Social support	Mental health
	B (*t*)	B (*t*)
Hardiness	0.71(17.94^***^)	0.46(10.85^***^)
Social support		0.41(10.92^***^)
Only child and Non-only child		−0.54(−2.03^*^)
Social support ^*^ only child and non-only child		0.14(2.16^*^)
*R^2^*	0.32	0.50
*F*	321.75^***^	167.09^***^

*B represents unstandardized coefficients*.

* *p < 0.05*;

*** *p < 0.001.*

## Discussion

This study developed a moderated mediation model to explore the internal mechanisms of the effect of hardiness on the mental health of Chinese FCSs. This study also examined the mediating role of social support and the moderating role of OC/NOC status in the aforementioned effect. The findings of this study suggest that hardiness affects the mental health of FCSs through social support and that the OC/NOC status moderates the second half of this mediating relationship. In addition, the OC/NOC status has a moderating effect on the relationship between social support and mental health.

### The Protective Role of Hardiness and Social Support in Mental Health

The study verified the mechanism of hardiness on the mental health among the specific groups of Chinese FCSs. According to the hardiness model, hardiness acts as a protecting factor for mental health (Hystad et al., 2009; Ramzi and Besharat, 2010; Yong, 2017; Sadeghi and Einaky, 2020). The results of this study conducted among Chinese FCSs are consistent with previous studies on the general student population and support Hypothesis 1, indicating that hardiness positively predicts the mental health of FCSs. The financial difficulties may burden them with more obstacles related to their lives, academic careers. Hardiness enables such students to adjust psychologically to changes in their environment and maintain a healthy mental state.

Meantime, the study explained how hardiness affects FCSs’ mental health by suggesting social support as key mediating variable, highlighting the importance of social support. The results support the hardiness model and match the main effect model of social support, indicating social support influences individual mental health directly (Barrera, 1986; Wang, 2004). The findings are consistent with previous studies (Du and Chai, 2014; Yang, 2016; Zhang et al., 2018) and support Hypothesis 2, exhibiting social support is another protecting factor for FCSs by acting mediating role in the relationship between hardiness and mental health. Hardy FCSs can receive and perceive more social support and utilize social resources to overcome difficulties in their studies. Moreover, hardiness can mitigate the negative effects of stress and help students maintain their mental health. For students in poverty, the society support is very crucial by alleviating the financial stress and enhancing their perception of social support to maintain good mental health.

### Moderating Role of OC/NOC Status

The study also explored the moderating effect of OC/NOC status, as an aspect of family composition, on the relationship of social support and mental health, which has not been studied in previous research. From the perspective of different treatment by parents, the psychological states, adjustment, and behavior of OCs or NOCs are diverse (Falbo and Polit, 1986; Kolm and Ythier, 2006; Cameron et al., 2013). The family may be an important contextual factor for FCSs on their perceived social support and mental health. Therefore, this study considered the OC/NOC status as moderating factors in the mediation model. The results find that social support had a stronger effect on the mental health of OCs compared to NOCs. It is consistent with previous studies and support Hypothesis 3, thus, the OC/NOC status moderates the relationship between social support and mental health among FCSs. One possible reason is the resource, which is already limited in FCSs’ families, is allocated more to OCs than NOCs, leading FCSs without siblings perceive more social support.

## Limitations

First, this study is limited by its sampling conditions. Questionnaires were distributed to the FCSs from a university in Yunnan province, in which the ratio of male and female students is approximately 1:3. It leads to the inequality of gender composition in samples. Future studies should consider expanding the geographical scope of sampling or to duplicate and verify our results in different colleges. Second, this study only took a cross-sectional quantity survey on the mental health of the FCSs. In-depth interviews or longitudinal studies could be conducted in the future. Third, the study only conducted among the FCSs group without comparison with the regular students or no-funded students, which could be designed in the future studies. Fourth, this study only explored whether the OC/NOC status moderated the second half of the mediating model pathway. Thus, future studies can explore whether this moderation effect exists in other pathways.

## Conclusion and Suggestions

This study explored the mediators and moderators of the effect of hardiness on the mental health of FCSs. It found that hardiness directly affects the mental health of such students and also indirectly affects their mental health through the mediating role of social support. Moreover, the results of this study indicated that the OC/NOC status moderated the mediating effect of social support on mental health, which indicates that the indirect effect of hardiness on mental health through social support was moderated by the OC/NOC status. Among the recruited students, social support had a stronger effect on the mental health of those are OCs than on the mental health of those who are NOCs. On the basis of the findings of this study, the following recommendations are proposed as:

1. Colleges should cultivate positive personality traits among FCSs and help them dismiss psychological poverty while providing economic support. College students should be instructed to increase their self-confidence and advance themselves while receiving social funding. They should also receive suitable guidance to enable them to face difficulties and challenges with a positive attitude; increase their self-control, psychological tolerance, and resilience; understand, adjust, and control themselves suitably; and process stress healthily instead of passively accepting it. In addition, FCSs should participate in social activities, solve problems in these activities, and learn to support themselves. Moreover, they should increase their hardiness to maintain a healthy mindset.
2. Colleges should introduce more sources of financial support for college students with economic difficulties to help them obtain social support and process stress. Financial support should be expanded from direct economic assistance to encourage society to provide employment and support to FCSs. With new employment opportunities, FCSs can transition from school to work easily. Such students should also be taught gratitude and imbued with an optimistic outlook and positive attitude to ensure that they appreciate the social support that they receive, utilize social resources to solve their problems, relieve stress, and maintain their mental health.
3. The results of this study found that social support had different effects on the mental health of OCs or NOCs. And it revealed that social support has a stronger effect on the mental health of FCSs who are OCs than on the mental health of those who are NOCs. Colleges should pay more attention to the educational and personal lives of FCSs who are NOCs, provide life guidance to them, and encourage them in their studies to help them process stress and thus improve their mental health.

## Data Availability Statement

Other data pertaining to this study are available from the corresponding author upon reasonable request.

## Ethics Statement

Ethical review and approval were not required for the study on human participants in accordance with the local legislation and institutional requirements. The patients/participants provided their written informed consent to participate in this study.

## Author Contributions

JLiu was the primary author who proposed the research proposal and completed the article for this study. XC and JLi worked as investigators and writer’s assistants. JLi served as the research advisor. The manuscript was revised collaboratively by JLiu, XC, and JLi. All authors contributed to the article and approved the submitted version.

## Conflict of Interest

The authors declare that the research was conducted in the absence of any commercial or financial relationships that could be construed as a potential conflict of interest.

## Publisher’s Note

All claims expressed in this article are solely those of the authors and do not necessarily represent those of their affiliated organizations, or those of the publisher, the editors and the reviewers. Any product that may be evaluated in this article, or claim that may be made by its manufacturer, is not guaranteed or endorsed by the publisher.
